# Feasibility of virtual reality-based simulation for neonatal resuscitation training: a pilot study at an international site

**DOI:** 10.1038/s41372-025-02382-2

**Published:** 2025-08-20

**Authors:** GiaKhanh Trinh, Binh Ho T. T, Jens C. Eickhoff, Ryan M. McAdams

**Affiliations:** 1 Department of Pediatrics, Altru Hospital, Grand Forks, ND USA; 2 Department of Neonatology, City Children’s Hospital, Ho Chi Minh City, Vietnam; 3 Department of Pediatrics, University of Wisconsin School of Medicine and Public Health, Madison, WI USA

**Keywords:** Developing world, Patient education

## Abstract

**Background:**

Virtual reality (VR) offers immersive training experiences that can address limitations of traditional neonatal resuscitation training. Building on prior U.S.-based research, we evaluated the feasibility of deploying a VR-based neonatal resuscitation model in an international setting.

**Methods:**

Healthcare providers at Ho Chi Minh City Children’s Hospital in Vietnam participated in a single-arm feasibility study using VR-based training. Sessions employed a team-based approach with physicians, nurses, and allied health professionals and included knowledge review, platform orientation, and a simulated resuscitation scenario based on the Neonatal Resuscitation Program. The simulation was delivered in English with real-time Vietnamese translation. Participants completed post-training surveys assessing satisfaction, realism, and challenges.

**Results:**

Among 28 participants, 100% recommended VR training; 86% found it more realistic than traditional methods. The mean usefulness score was 4.3/5. Challenges included language barriers and technical issues.

**Conclusions:**

VR-based neonatal resuscitation training is feasible and well-received at Ho Chi Minh City Children’s Hospital, Vietnam.

**Clinical Trial Registration:**

Not applicable.

## Introduction

Neonatal resuscitation is a critical skill for healthcare providers who attend newborn deliveries, as approximately 10% of newborns require assistance to initiate breathing at birth [1]. Effective resuscitation in these high-pressure scenarios significantly impacts immediate and long-term neonatal outcomes [2–4]. Consequently, ensuring that healthcare providers possess the necessary competencies is essential, with simulation-based training forming the cornerstone of education in neonatal resuscitation programs worldwide [5].

Traditional training methods, including manikin-based simulations, have proven effective but are not without limitations. High-fidelity manikins, while valuable, struggle to fully replicate the immersive, variable, and dynamic nature of real-life clinical scenarios. Additionally, the high cost, space requirements, and resource-intensive setup of these simulations limit their accessibility, especially in resource-constrained settings. These barriers underscore the need for innovative approaches to complement or, in some cases, replace traditional methods.

Virtual reality (VR), augmented reality, and mixed reality have emerged as transformative tools in medical education, offering equal knowledge acquisition compared to traditional methods while significantly enhancing learner satisfaction, engagement, and self-efficacy. [6] VR enhances neonatal resuscitation training by providing immersive, customizable simulations that improve procedural skills, decision-making, and learner confidence while offering a scalable and accessible solution for diverse healthcare settings [7]. A systematic review by Kyaw et al. demonstrated that VR improves post-intervention knowledge retention and cognitive skill acquisition, with a large effect size (Standardized Mean Difference = 1.12; 95% CI 0.81–1.43) [8]. This evidence underscores VR’s capacity to simulate procedural and decision-making tasks effectively, making it a valuable tool for training healthcare professionals in high-stakes settings like neonatal resuscitation. However, the application of VR in neonatology remains underexplored, with limited studies evaluating its effectiveness, feasibility, and scalability across diverse cultural and healthcare settings.

Building on prior research conducted in the United States (U.S.) [9], this study sought to extend the use of VR-based neonatal resuscitation training to an international context, specifically in Ho Chi Minh City, Vietnam. The objectives were to assess the feasibility, acceptance, and challenges of implementing this training model in a resource-constrained setting and to identify areas for refinement to improve its effectiveness. A key methodological adaptation for this international implementation was the adoption of a team-based training model with enhanced nursing and allied health representation. This approach was designed to better reflect the interdisciplinary team structure characteristic of traditional NRP education, where effective neonatal resuscitation relies on coordinated efforts among physicians, nurses, respiratory therapists, and other healthcare providers. By incorporating team-based dynamics into the VR environment, this study aimed to explore the feasibility of delivering interdisciplinary training and to assess participant perceptions of how the platform may support collaborative decision-making, role clarity, and communication skills essential for effective neonatal resuscitation.

## Methods

### Study design, setting, and participants

This cross-sectional, single-arm pilot study evaluated the feasibility and participant reception of a VR-based neonatal resuscitation training program in Ho Chi Minh City, Vietnam; as there was no control group, the study focused on implementation and user experience rather than comparative effectiveness. The study was conducted at the Neonatal Intensive Care Unit (NICU) of Ho Chi Minh City Children’s Hospital, a tertiary care center serving a diverse patient population. Participants included healthcare providers involved in neonatal care, recruited via in-person announcements and electronic communication. Inclusion criteria were active clinical involvement in neonatal resuscitation and willingness to participate in the study.

A total of 28 healthcare providers participated, including neonatologists, pediatric residents, nurses, and allied health professionals. Written informed consent was obtained from all participants prior to the study. Ethical approval was secured from the Institutional Review Board of the University of Wisconsin-Madison and the local ethics committee in Vietnam.

### Virtual reality model development

The VR training platform was developed using Acadicus (Arch Virtual, Madison, WI, USA) in collaboration with a multidisciplinary team of neonatologists, VR developers, and simulation experts. The training model simulated a 30-week neonate experiencing respiratory distress, with scenarios aligned to the Neonatal Resuscitation Program® (NRP) 8th edition guidelines [10]. The virtual neonate exhibited key clinical features, including chest rise, retractions, and changes in vital signs, providing real-time dynamic feedback during the resuscitation scenario.

**Fig. 1:**
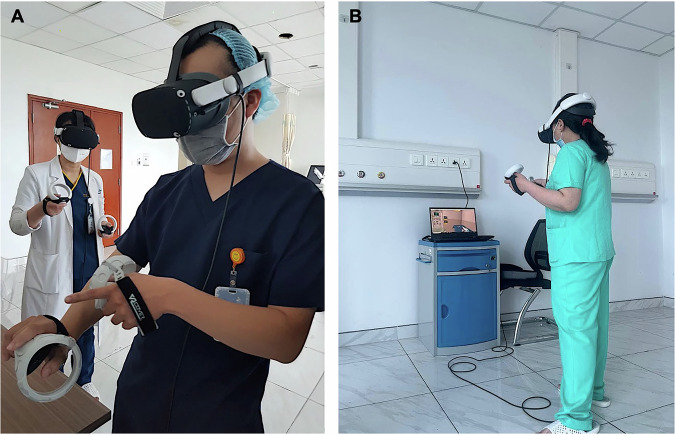
Healthcare providers at Ho Chi Minh City Children’s Hospital participating in virtual reality (VR)-based neonatal resuscitation training. **A** Two participants wear VR head-mounted displays and handheld controllers, engaging in simulated resuscitation scenarios designed to reinforce neonatal resuscitation protocols. **B** A participant navigates the VR simulation while a nearby laptop displays the virtual environment for facilitator observation and feedback. The VR-based platform offers immersive, interactive training to enhance cognitive and team-based skills in neonatal resuscitation.

Participants engaged with the virtual environment using the Meta Quest 2 head-mounted displays (Meta Platforms, Inc., Menlo Park, CA, USA) and handheld controllers, as shown in Fig. 1. The training maintained consistency with our previously published U.S. pilot study [9], while incorporating adaptations to accommodate local contextual factors in Vietnam. A significant methodological evolution from our previous individual-focused VR training study was the deliberate adoption of a group-based training approach with enhanced nursing and allied health representation. This team-based model was identified as a critical feature to explore based on the recognition that traditional NRP education emphasizes interdisciplinary collaboration, and effective neonatal resuscitation inherently requires coordinated teamwork among diverse healthcare professionals. This study utilized a group-based training approach with enhanced nursing representation, allowing nurses and allied health professionals to train alongside physicians, mirroring the team-based structure of traditional NRP education.

The training environment was designed as a fully equipped virtual resuscitation room, providing participants with an immersive and interactive experience. Within this environment, participants were guided through essential tasks such as mask ventilation, implementing corrective steps, and providing post-resuscitation care. The corrective steps, known as MR. SOPA, include specific interventions to improve ventilation: Mask adjustment, repositioning the airway, suctioning the mouth and nose, opening the mouth, increasing Pressure, and using an Alternative airway if necessary. The VR platform featured pre-recorded instructions that facilitated navigation and interaction with virtual equipment, allowing participants to complete key steps in the resuscitation process.

### Intervention

The training sessions were conducted over two weeks in April 2023 and consisted of four group sessions, each involving 6–8 participants. Each session followed a structured format with four key components. The first component was an introduction and orientation led by a study team member and co-author (G.T.), who was based at the University of Wisconsin–Madison but was onsite in Vietnam for the duration of the study. G.T. facilitated the sessions, providing an overview of the training objectives and logistics, and utilized her fluency in Vietnamese to ensure effective communication with participants. To further support the sessions, an onsite team member assisted with translation and technical troubleshooting.

The second component was a virtual environment orientation module. Participants completed a guided walkthrough to familiarize themselves with the VR navigation tools and virtual equipment. This step was designed to build their confidence and competence before entering the simulated scenario.

**Fig. 2:**
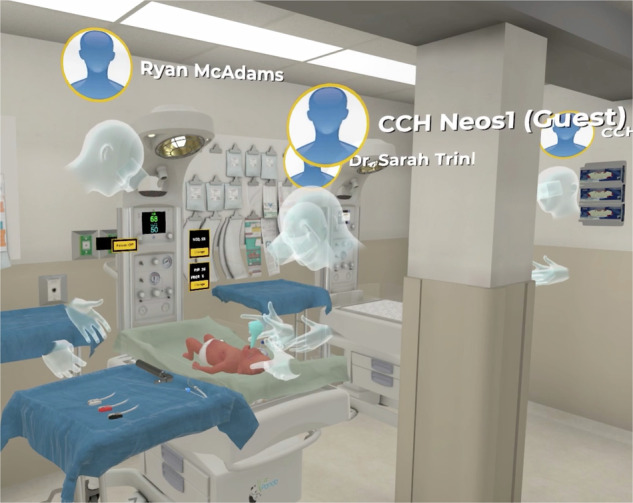
Screenshot of the virtual reality (VR) neonatal resuscitation training environment used in the pilot study at Ho Chi Minh City Children’s Hospital. The immersive scenario depicts a simulated neonatal resuscitation room with a preterm infant on a radiant warmer. Participants, represented by avatars, are shown performing bulb suctioning of the infant’s mouth as part of the MR. SOPA algorithm for ventilation corrective steps. During this session, Vietnamese healthcare provider participants were joined by co-author R.M., located remotely in Madison, Wisconsin, and co-author G.T., who was onsite in Ho Chi Minh City, providing real-time translation of R.M.’s instructions. The VR platform enabled interdisciplinary and international collaboration, simulating neonatal resuscitation procedures in accordance with the Neonatal Resuscitation Program®. Interactive equipment, including a resuscitation warmer, ventilation devices, and laryngoscope tools, allowed participants to practice cognitive decision-making and team-based communication skills in a realistic, immersive, and safe training space.

The third component involved the simulation scenario. With each training session, a remote study team member and co-author (R.M.), based at the University of Wisconsin–Madison, greeted participants via Zoom® (Zoom Video Communications, Inc., San Jose, CA) and subsequently joined them in the virtual environment to guide the simulation scenario. Participants managed a case of a 30-week neonate with respiratory distress. Within the virtual environment (Fig. 2), participants performed critical MR. SOPA corrective steps as illustrated in the supplemental video (Appendix). The scenario, aligned with the NRP algorithm, included pre-programmed clinical changes that required participants to perform critical interventions such as positive pressure ventilation and corrective actions.

The fourth component was a debriefing and feedback session. During this concluding phase, participants engaged in a facilitated discussion to reflect on their performance, share insights, and receive constructive feedback. Following the debriefing, participants completed a post-training survey to evaluate the session’s effectiveness and provide suggestions for improvement.

To accommodate the international setting and remote instruction model, training utilized a bilingual approach with real-time translation support. Approximately 25% of the training was delivered in English with Vietnamese translation, while 75% was conducted in Vietnamese. The theoretical knowledge review was delivered in English by the U.S.-based instructor (R.M.) with simultaneous Vietnamese translation, while simulation scenarios were conducted primarily in Vietnamese to facilitate natural team communication and reduce cognitive load. The VR system interface and pre-recorded instructional content were English-only, requiring human translation throughout the sessions. A bilingual study team member (G.T.), who was also a certified NRP instructor, provided all translation services and technical coordination between the remote U.S. instructor and local participants. This translation approach was necessary to enable real-time remote instruction from the U.S.-based instructor who possessed specific technical expertise with the VR platform while ensuring effective communication with Vietnamese-speaking participants.

### Survey development

The post-training survey used in this study was adapted from the survey developed for our prior U.S. study in collaboration with the University of Wisconsin Survey Center [9]. To ensure linguistic and cultural accuracy, the survey underwent a two-step translation process. It was first translated from English to Vietnamese by professional translators recommended by UW-Madison, followed by an independent back-translation into English to verify accuracy (see Appendix for the survey tool). A team member (G.T.) then conducted a final review to refine linguistic precision and cultural relevance.

The survey was administered electronically using the Qualtrics platform (Qualtrics, Provo, UT) and assessed primary outcomes, including participant satisfaction, perceived realism, and the usefulness of the VR model. Secondary outcomes included reports of adverse effects associated with VR use (e.g., dizziness, nausea) and implementation challenges. Additionally, open-ended questions captured qualitative feedback for thematic analysis, providing deeper insight into participant experiences and areas for improvement.

### Data analysis

Quantitative data were analyzed using descriptive statistics, with categorical variables presented as frequencies and percentages and continuous variables as means with standard deviations. Subgroup analyses were performed to examine differences in VR ratings by role, prior VR experience, and years of NICU experience. Comparative analysis with the prior U.S. study was conducted using chi-square or t-tests as appropriate. Qualitative data from open-ended survey responses were analyzed using thematic analysis to identify recurring themes and subthemes following the approach described by Braun and Clarke [11].

## Results

### Participant demographics

A total of 28 healthcare providers participated in the VR-based neonatal resuscitation training in Vietnam (Table 1). Participants included pediatric residents (11%), NICU hospitalists (29%), neonatologists (4%), nurses (18%), and other healthcare professionals (18%). Most participants were female (70%) and had 3–5 years of NICU experience (39%), while 28% had less than three years of NICU experience. Only 22% of participants had completed the American NRP course, highlighting a relative lack of formal resuscitation training within the cohort.

**Table 1:** Participant Demographics (Vietnam Cohort).

Demographic Variable	N (%)
**Role**	
Pediatric Resident	3 (11%)
NICU Hospitalist	8 (29%)
Neonatologist	1 (4%)
Nurse	5 (18%)
Other	11 (39%)
**Gender**	
Female	20 (70%)
Male	8 (30%)
**Years of NICU Experience**	
Less than 3 years	8 (28%)
3–5 years	11 (39%)
More than 5 years	9 (33%)
**Prior VR Exposure**	
Had used VR before	9 (32%)
Owned a VR headset	5 (18%)

Summary of the professional roles, gender distribution, years of NICU experience, and prior virtual reality (VR) exposure among 28 healthcare providers who participated in the VR-based neonatal resuscitation training in Ho Chi Minh City, Vietnam.

### VR familiarity and training experience

Prior VR exposure was limited among participants, with only 32% reporting prior use of a VR headset, and 18% owning one (Table 1). All participants successfully completed the VR orientation module and simulation scenario, with 79% indicating a desire to revisit the orientation module before future sessions. The training program included a combination of virtual walkthroughs, simulation exercises, and debriefing, ensuring a structured and immersive learning experience.

### Satisfaction and perceived usefulness

Participant satisfaction with the VR training was positive. All participants (100%) stated they would recommend VR training to colleagues, and 86% rated the VR simulation as more realistic than traditional training methods. The average usefulness score for the VR platform was 4.3 out of 5 (standard deviation: 0.9), reflecting high perceived value among users (Table 2).

**Table 2:** Statistical Comparison of Key Outcomes.

Group	Mean Usefulness Score (SD)	*P*-Value
**Role**		
Pediatric Resident	4.3 (0.6)	0.0728*
NICU Hospitalist	4.3 (0.5)	
Neonatologist	1.0 (0.0)	
Nurse	4.0 (1.2)	
Other	4.7 (0.5)	
**NICU Experience**		
Less than 3 years	4.2 (1.3)	0.8753
3–5 years	4.1 (0.4)	
More than 5 years	4.0 (0.5)	

Comparison of average usefulness scores across participant subgroups stratified by professional role and NICU experience. *P*-values indicate the significance of observed differences.

*P-value: *p*-value for comparison of Neonatologist/NICU Hosp/Nurse vs. all others combined.

### Comparison with U.S. cohort

Compared to a prior U.S.-based cohort (Table 3), the Vietnamese participants reported higher recommendation rates (100% vs. 95%) and higher perceived realism (86% vs. 70.3%). Furthermore, VR-related discomfort, such as dizziness or nausea, was reported by only 28.6% of Vietnamese participants, compared to 40.5% of the U.S. cohort.

**Table 3:** Comparison of feedback between U.S. and Vietnam Cohorts.

Feedback Metric	U.S. Cohort (*N* = 38)	Vietnam Cohort (*N* = 28)	*P*-value
Would recommend VR training to colleagues	95%	100%	0.5040
Found VR more realistic than traditional methods	70.3%	86%	0.1306
Reported VR-related discomfort	40.5%	28.6%	0.3839
Average usefulness score (Likert 1–5), mean (SD)	4.5 (0.8)	4.3 (0.9)	0.3447

Comparison of participant feedback on virtual reality (VR) training between cohorts from the U.S. and Vietnam, including rates of recommendation, perceived realism, reported VR-related discomfort, and average usefulness scores. *P*-values indicate statistical differences between groups.

### Challenges and qualitative feedback

Participants identified several challenges during the training, including unfamiliarity with the NRP algorithm (78%, or 22 out of 28 participants had not completed the American NRP course), intermittent internet connectivity (9%, or 2 out of 22 qualitative responses), and language barriers that required onsite translation support. Despite these obstacles, participants expressed appreciation for the novelty and potential of the VR platform.

Thematic analysis of open-ended survey responses revealed key areas for improvement, including a desire for greater scenario realism and clinical variability (36%, or 8 out of 22 qualitative responses), increased opportunities for practice and experience with VR technology (23%, or 5 out of 22 qualitative responses), and improved technological infrastructure. Additionally, participants valued the teamwork fostered through VR training and emphasized the importance of safe practice environments that do not pose risks to real patients.

## Discussion

This study demonstrates the feasibility and positive reception of a VR-based neonatal resuscitation training program in an international setting, specifically in Vietnam. Through systematic coding and theme development, this analysis identified patterns that highlight both the challenges and opportunities in VR-based training implementation. Building on prior research conducted in the U.S. [9], this pilot study confirms the adaptability of the VR platform across diverse healthcare environments and highlights its potential as an innovative training tool for neonatal resuscitation.

### Comparison with traditional training methods

The Vietnamese participants reported high satisfaction with the VR training, with 86% rating it as more realistic than traditional training methods. This aligns with previous findings that VR offers an immersive learning environment, enabling repeated exposure to high-stakes clinical scenarios in ways that traditional manikin-based training may not, though some learners perceive limitations in realism and patient interaction [12]. The dynamic and interactive nature of the VR platform allowed participants to engage with a simulated 30-week neonate, providing valuable opportunities to practice critical interventions such as positive pressure ventilation and corrective steps. These features are particularly relevant in settings with limited access to high-fidelity manikins or formal neonatal resuscitation training.

A key distinction between this study and our previous VR-based neonatal resuscitation training study [9] was the shift from an individualized to a team-based training approach with enhanced nursing and allied health representation. As established in our methodology, this study was designed to better reflect real-world clinical team dynamics and align with the interdisciplinary structure of traditional NRP education. By allowing nurses, respiratory therapists, and other allied health providers to train alongside physicians, the VR sessions enhanced the realism of the simulation and underscored the essential role of coordinated teamwork in neonatal resuscitation. This approach may better prepare participants for clinical practice by promoting collaborative decision-making, clarifying team roles, and strengthening communication skills—all of which are critical for optimizing neonatal outcomes. These findings suggest that VR-based training can be effectively expanded beyond individual skill acquisition to enhance team dynamics, an aspect that warrants further exploration in future studies.

### International adaptability and cultural considerations

This study highlights the international adaptability of VR-based training. Compared to the U.S. cohort, the Vietnamese participants expressed higher perceived realism and comfort with the VR platform, despite lower baseline familiarity with VR technology. This suggests that VR can potentially bridge gaps in training quality in resource-constrained settings. However, unique challenges were encountered, including language barriers and limited familiarity with the NRP algorithm. Future iterations of the program should incorporate bilingual instruction and region-specific clinical guidelines to further enhance accessibility and relevance.

### Implementation challenges and potential improvements

Despite the overwhelmingly positive reception, several implementation challenges were noted. Language implementation required real-time human translation, as the VR system lacked built-in multilingual capabilities. While effective, this approach necessitated dedicated translator presence and added logistical complexity.

Technical infrastructure also presented challenges, particularly regarding internet connectivity. Unstable internet connections occasionally disrupted the immersive experience, as stable connectivity was required to enable real-time communication between local participants and the remote U.S.-based instructor (R.M.) who joined the virtual environment simultaneously. Initially, the hospital’s internal Wi-Fi network encountered significant latency issues, which were resolved by using individual mobile hotspots for each VR station and connecting headsets via physical cables to reduce wireless dependency.

Additionally, participants expressed a desire for increased scenario variability and more opportunities for practice to enhance skill mastery. Future implementations would benefit from VR systems with integrated language selection and localized content, improved technical infrastructure, and expanded scenario libraries to enhance scalability and effectiveness.

### Virtual environment familiarity and adaptation

One important consideration when deploying VR-based neonatal resuscitation training is the potential adjustment period required for participants to familiarize themselves with a virtual environment that may differ from their actual clinical setting. The layout of virtual resuscitation rooms, positioning of equipment, and user interfaces may not precisely mirror participants’ real-world work environments. This discrepancy could lead to an initial learning curve, requiring additional time to orient participants to the virtual space before they can fully engage in resuscitation tasks. Future research should evaluate the extent to which this familiarization process impacts training efficiency, skill acquisition, and overall learning outcomes. Optimizing the design of virtual environments to reflect local clinical settings may help minimize disorientation and enhance the realism and relevance of the simulation experience.

### Flexibility to accommodate multiple resuscitation algorithms

While the VR training platform in this study was designed according to the NRP 8th edition guidelines, there are several neonatal resuscitation algorithms currently in use across different regions and institutions. A key advantage of VR technology is its adaptability; virtual scenarios can be tailored to reflect various protocols and guidelines, thereby supporting widespread applicability. However, certain procedural elements—such as endotracheal intubation and chest compressions—may currently lack the tactile realism provided by advanced haptic feedback systems, which remain in early stages of development. Despite this limitation, VR offers an effective modality for practicing cognitive elements of neonatal resuscitation, including algorithm adherence, clinical decision-making, and team coordination.

### Psychological impact of training in a virtual environment

Another important consideration is the psychological experience of participants during VR-based simulations. Ideally, these environments promote psychological safety so learners can engage fully, take risks, and learn from mistakes without fear of embarrassment or judgment. This sense of safety supports critical behaviors such as asking questions, reflecting on performance, and openly acknowledging uncertainty—behaviors that are foundational to simulation-based education [13]. Psychological safety is especially important in unfamiliar learning environments like VR, where the novelty of the interface can heighten anxiety. Effective strategies to foster this safety begin before the simulation itself. A structured pre-briefing that clarifies learning objectives, roles, expectations, and evaluation methods can help reduce ambiguity and support learner engagement. As described by Rudolph et al., the pre-briefing also establishes a “fiction contract,” a mutual agreement that acknowledges the simulation’s limitations while encouraging learners to interact with the scenario as if it were real [14]. This contract normalizes performance variability and invites participants to focus on learning rather than scenario appearance. In VR contexts, where the simulated experience may feel isolating or immersive in unfamiliar ways, facilitator support plays an important role in encouraging curiosity and demonstrating respect for the learner’s perspective. Future research should explore how psychological safety influences learning outcomes in VR-based training and identify effective, culturally adaptable strategies for creating safe learning environments across diverse settings.

### Cybersickness and VR-related discomfort across cohorts

While VR can serve as a valuable adjunct to traditional manikin-based neonatal resuscitation simulations, challenges such as cybersickness warrant consideration. Cybersickness, which is characterized by symptoms like dizziness, nausea, and disorientation, is a frequently reported side effect of VR use [15, 16]. Previous studies have shown that a significant proportion of users experience these symptoms, with rates as high as 49% among midwifery students [17] and 23% among medical students receiving VR-based trauma education [18]. In our study, 28.6% of participants reported VR-related discomfort, a lower incidence compared to our U.S. cohort (40.5%) [9], yet still notable. This reduction may reflect differences in session structure, participant demographics, or prior VR exposure. Regardless, the overall occurrence of discomfort highlights the need for mitigation strategies, such as improved hardware, stable internet connectivity, and structured orientation. Strategies such as limiting session lengths to one hour, as recommended by Holla and Berg [18], and incorporating gradual acclimation to the VR environment could potentially mitigate these effects. In addition to motion sickness, prolonged VR use may lead to oculomotor strain, although both symptoms may diminish with repeated exposure [19]. Addressing these challenges through user preparation and technological refinements may help optimize the effectiveness and accessibility VR-based training programs. Further research is needed to understand the contributing factors to cybersickness and oculomotor strain, including how these issues vary across educational backgrounds and experience levels.

### Strengths and limitations

A major strength of this study is its demonstration of VR training’s feasibility in a resource-limited international setting. The study design allowed for structured evaluation of participant attitudes and challenges, providing valuable insights for future adaptations. However, several limitations warrant discussion. The reliance on self-reported survey data introduces potential response bias, as participants may have been inclined to provide favorable feedback. In addition, the absence of a control group limits the ability to directly compare VR training outcomes with those of traditional methods. This pilot study also introduced multiple design variables simultaneously, including an international site, bilingual delivery, remote facilitation, and a team-based training model. While these features reflect the practical realities of global implementation, they limit our ability to isolate the individual contributions of each factor to the training outcomes. Future studies should consider more controlled designs to examine the specific effects of each component. Finally, the small sample size and limited representation of certain professional roles, such as nurses and allied health professionals, may affect the generalizability of the findings.

### Future directions

Future research should focus on addressing identified challenges and expanding the scope of evaluation. Comparative studies with larger, more diverse samples are needed to rigorously assess the effectiveness of VR training relative to traditional methods. Objective outcome measures, such as skill retention and clinical performance, should complement participant feedback to provide a more comprehensive understanding of the program’s impact. Additionally, integration of emerging technologies, such as artificial intelligence, could enhance the adaptability and interactivity of VR-based training. Features such as real-time feedback, automated performance assessment, and multilingual support have the potential to address current limitations and further optimize training outcomes.

## Conclusion

This pilot study demonstrates that VR-based neonatal resuscitation training is a feasible, effective, and well-received approach in a hospital in Vietnam. By providing immersive, realistic, and accessible training experiences, VR has the potential to transform neonatal resuscitation education, particularly in resource-limited contexts. Addressing implementation challenges and conducting rigorous evaluations will be critical to unlocking the full potential of this innovative training tool.

## Supplementary information

Virtual reality neonatal resuscitation simulation video

Survey Tool

## Data Availability

The datasets generated and analyzed during the current study consist of survey responses and are not publicly available due to participant confidentiality, but de-identified data may be available from the corresponding author upon reasonable request.
