# Supplementary material for: Feasibility of virtual reality-based simulation for neonatal resuscitation training: a pilot study at an international site
Source: J Perinatol. 2025 Aug 20;45(11):1579–85. doi: 10.1038/s41372-025-02382-2 (PMC12660134; doi:10.1038/s41372-025-02382-2)
Supplement: Supplementary file 2 — Survey Tool [file 41372_2025_2382_MOESM2_ESM.pdf]

Survey (Translated from Vietnamese into English)

You are invited to participate in the "Virtual Reality Distance Simulation - A Novel Educational Tool For Neonatal Resuscitation Training In A Middle-Income Country" project, conducted by Dr. Sarah Trinh and Dr. Ryan McAdams, Division of Neonatology, University of Wisconsin-Madison and Dr. Binh Ho, Department of Neonatology, Ho Chi Minh City Children's Hospital. The purpose of this project is to assess the role of Virtual Reality in Distance simulation for Neonatal Resuscitation Training.

If you choose to be in the study, we will ask you to complete a survey. The survey will help us learn more about topic(s). The survey will take about 5 minutes for you to complete. You can skip any survey questions that you do not want to answer. Even if you start the survey, you are not required to complete it. You can stop at any time. All your answers will be confidential and will not be shared with anyone outside the research team. The survey is anonymous, and no one will be able to link your answers back to you. Please do not include your name or other information that could be used to identify you in the survey responses.

If you have any questions about the project, please contact Dr. Sarah Trinh (ntrinh@uwhealth.org). This QI project has been reviewed according to the University of Wisconsin Health Sciences IRB procedures for Exempted Projects.

ELECTRONIC CONSENT: Please select your choice below.

Clicking on the "agree" button below indicates that:

- You have read the above information
- You voluntarily agree to participate
- You are at least 18 years of age

If you do not wish to participate in the QI project, please decline participation by clicking on the "disagree" button.

○ Agree

○ Disagree

Familiarity with VR

The first questions are about your general experiences with virtual reality, also known as VR.

Had you ever used a VR headset before completing the Virtual Reality Neonatal Resuscitation Simulation Training Module?

- Yes
- No

Do you own a VR headset?

- Yes
- No

About how often do you use your VR headset?

- Once a week or more often
- 2 or 3 times a month
- Once a month
- Once every few months
- Once or twice a year, or less often

Module Feedback

The next questions are about your experience with the Virtual Reality Neonatal Resuscitation Simulation Training Module

Did you complete the Orientation module during this training session?

- Yes
- No

How useful was the Orientation module in helping you familiarize with the VR environment?

- Not at all useful
- A little usefu

- Somewhat useful
- Very useful
- Extremely useful

Would you want to view the Orientation module again prior to next VR training session?

- Yes
- No
- Unsure

What was the reason you did not complete the Orientation module?

- I am familiar with VR
- The module was not offered
- Other reasons

Please tell us about your reasons

Would you try VR simulation for neonatal resuscitation training again?

- Yes
- No
- Unsure

Does VR provide a more realistic experience compared to traditional neonatal resuscitation training?

- Yes
- No
- Unsure

Would you recommend VR simulation neonatal resuscitation training to others?

- Yes
- No
- Unsure

How useful do you think VR is as a tool for neonatal resuscitation training?

- Not at all useful
- A little useful
- Somewhat useful
- Very useful
- Extremely useful

What would make your VR training experience better?

Did you experience each of the following symptoms during or as a result of your VR training experience?

	Yes	Maybe	No
Headache	O	O	O
Dizziness	O	O	O
Eye strain	O	O	O
Other symptoms	O	O	O

Please tell us more about your symptoms

If you experienced any unwanted symptoms during your VR experience, how long did they last after you finished your session?

- I did not experience any symptoms

- Immediately after I finished
- Less than 5 minutes
- 5 to 10 minutes
- 10 to 15 minutes
- 15 to 20 minutes
- 20 to 30 minutes
- More than 30 minutes

Knowledge Assessment

The VR training module you experienced focused on corrective steps to initiate ventilation in a newborn. What does the MR SOPA acronym stand for?

M ____

R ____

S ____

O ____

P ____

A ____

Demographics

The last questions are about you.

What is your gender?

- Male
- Female

- Non-binary
- Prefer not to say

What is your current clinical role?

- Specialist Level II - Neonatologist
- Specialist Level I - Pediatrician
- Pediatric Resident or Specialist Level I trainee
- Interns - 18-month basic training
- NICU nurse
- Advanced Practice Provider
- Medical students
- Other

What is the main setting of your current practice? (Please select all that apply)

- [ ] Neonatal Intensive Care Unit
- [ ] Neonatal Ward
- [ ] Newborn Nursery
- [ ] Emergency Department
- [ ] Pediatric/Neonatal Clinics
- [ ] Surgery
- [ ] Pediatric ICU
- [ ] Other

How many years of NICU experience do you have?

- Less than 3 years
- 3 to 5 years
- 6 to 10 years
- More than 10 years

Do you have American Neonatal Resuscitation Program (NRP) Certificate?

- Yes
- No

In the last 5 years, how many Neonatal Resuscitation Program classes have you attended?

- None
- 1 - 2
- 3 - 5
- More than 5

In the last 5 years, did you attend any other Neonatal resuscitation training beside NRP?

- Yes
- No

If yes, please tell us more about the other neonatal resuscitation training you have attended

Comments

Do you have any other thoughts on the VR training experience you would like to share?

Powered by Qualtrics
